# Challenges in treatment of patients with non-classic congenital adrenal hyperplasia

**DOI:** 10.3389/fendo.2022.1064024

**Published:** 2022-12-12

**Authors:** Bas P. H. Adriaansen, Mariska A. M. Schröder, Paul N. Span, Fred C. G. J. Sweep, Antonius E. van Herwaarden, Hedi L. Claahsen-van der Grinten

**Affiliations:** ^1^ Radboud Institute of Health Sciences, Department of Laboratory Medicine, Radboud University Medical Center, Nijmegen, Netherlands; ^2^ Department of Pediatric Endocrinology, Amalia Children’s Hospital, Radboud University Medical Center, Nijmegen, Netherlands; ^3^ Radiotherapy & OncoImmunology Laboratory, Radboud Institute of Molecular Life Sciences, Department of Radiation Oncology, Radboud University Medical Center, Nijmegen, Netherlands

**Keywords:** Non-classic congenital adrenal hyperplasia (NCCAH), 21-hydroxylase deficiency (21OHD), 11-hydroxylase deficiency (11OHD), treatment options, glucocorticoid treatment

## Abstract

Congenital adrenal hyperplasia (CAH) due to 21α-hydroxylase deficiency (21OHD) or 11β-hydroxylase deficiency (11OHD) are congenital conditions with affected adrenal steroidogenesis. Patients with classic 21OHD and 11OHD have a (nearly) complete enzyme deficiency resulting in impaired cortisol synthesis. Elevated precursor steroids are shunted into the unaffected adrenal androgen synthesis pathway leading to elevated adrenal androgen concentrations in these patients. Classic patients are treated with glucocorticoid substitution to compensate for the low cortisol levels and to decrease elevated adrenal androgens levels *via* negative feedback on the pituitary gland. On the contrary, non-classic CAH (NCCAH) patients have more residual enzymatic activity and do generally not suffer from clinically relevant glucocorticoid deficiency. However, these patients may develop symptoms due to elevated adrenal androgen levels, which are most often less elevated compared to classic patients. Although glucocorticoid treatment can lower adrenal androgen production, the supraphysiological dosages also may have a negative impact on the cardiovascular system and bone health. Therefore, the benefit of glucocorticoid treatment is questionable. An individualized treatment plan is desirable as patients can present with various symptoms or may be asymptomatic. In this review, we discuss the advantages and disadvantages of different treatment options used in patients with NCCAH due to 21OHD and 11OHD.

## Introduction

**Figure 1:**
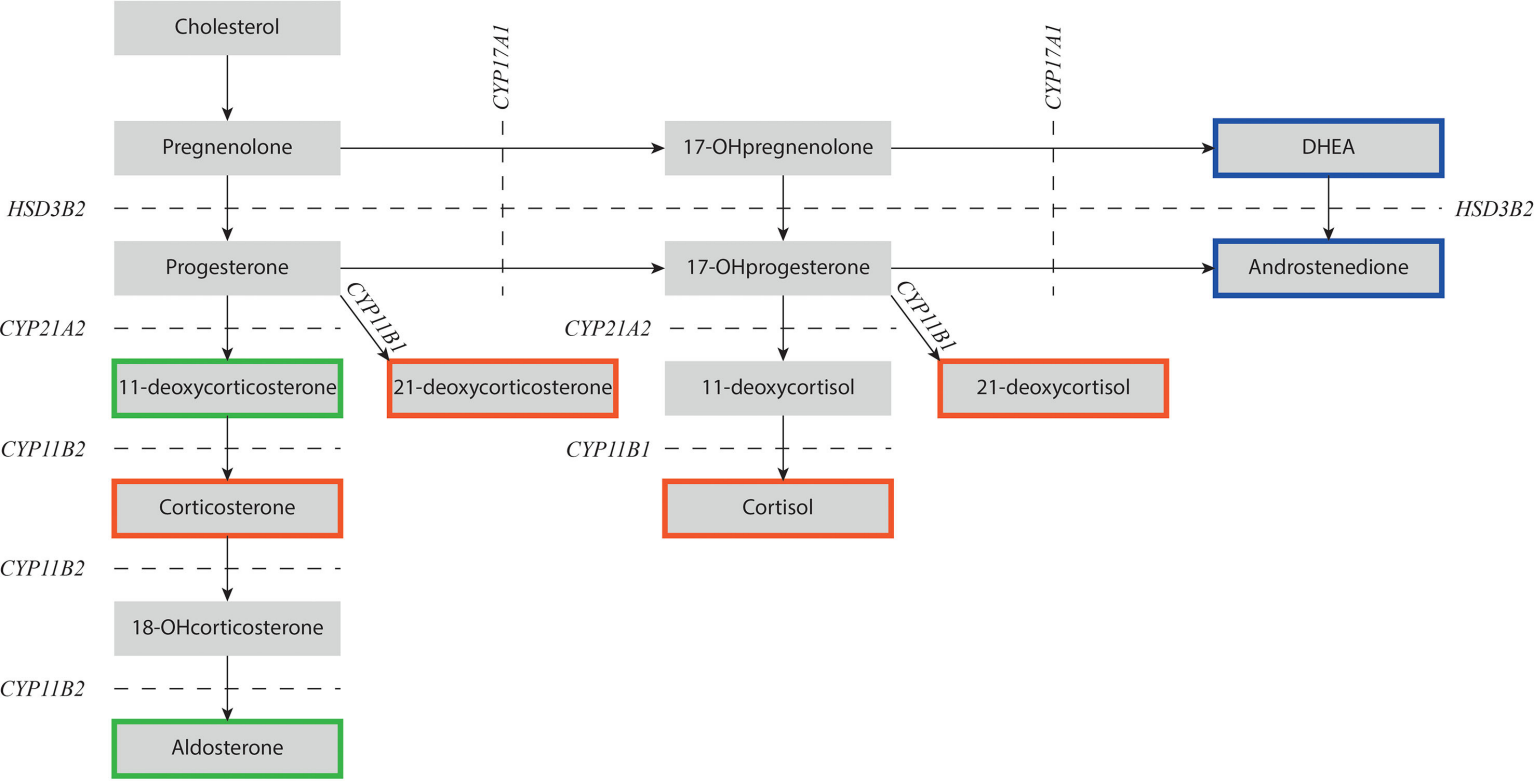
Schematic overview of adrenal steroidogenesis. NCCAH patients with 21OHD have impaired 21α-hydroxylase activity (*CYP21A2*) and NCCAH patients with 11OHD have impaired 11β-hydroxylase activity (*CYP11B1*). Precursor steroids prior to the enzymatic block increase and are shunted into androstenedione that can be converted into testosterone and dihydrotestosterone in the gonads. Steroids depicted in a red box have mainly glucocorticoid activity, steroids in a green box have mainly mineralocorticoid activity, and steroids in a blue box have mainly androgen activity. DHEA, dehydroepiandrosterone; *CYP11B1*, 11β-hydroxylase; *CYP11B2*, aldosterone synthase; *CYP17A1*, 17α-hydroxylase/17,20-lyase; *CYP21A2*, 21α-hydroxylase; *HSD3B2*, 3β-hydroxysteroid dehydrogenase type 2; OH-, hydroxy-.

Congenital adrenal hyperplasia (CAH) is a group of autosomal recessive disorders with affected adrenal steroidogenesis leading to impaired cortisol synthesis. Consequently, the production of adrenocorticotropic hormone (ACTH) is increased due to reduced negative feedback on the pituitary gland. In most cases, CAH is caused by a deficiency of 21α-hydroxylase (21OHD) (1). In more rare cases, CAH is due to a deficiency of other enzymes such as 11β-hydroxylase, 17α-hydroxylase, or 3β-hydroxysteroid dehydrogenase (2). The specific hallmark of 21OHD and 11OHD is impaired production of cortisol and an elevation of the adrenal androgen concentration. In patients with 21OHD, the conversion of 17-hydroxyprogesterone (17-OHP) to 11-deoxycortisol is impaired, resulting in elevated levels of 17-OHP that is metabolized into 21-deoxycortisol and adrenal androgens (**Figure 1**) (3). In patients with 11OHD, the conversion of 11-deoxycortisol to cortisol is impaired resulting in increased levels of 11-deoxycortisol and adrenal androgens (**Figure 1**).

In this review, we focus on the description of 21OHD with a short separate section about 11OHD.

**Figure 2:**
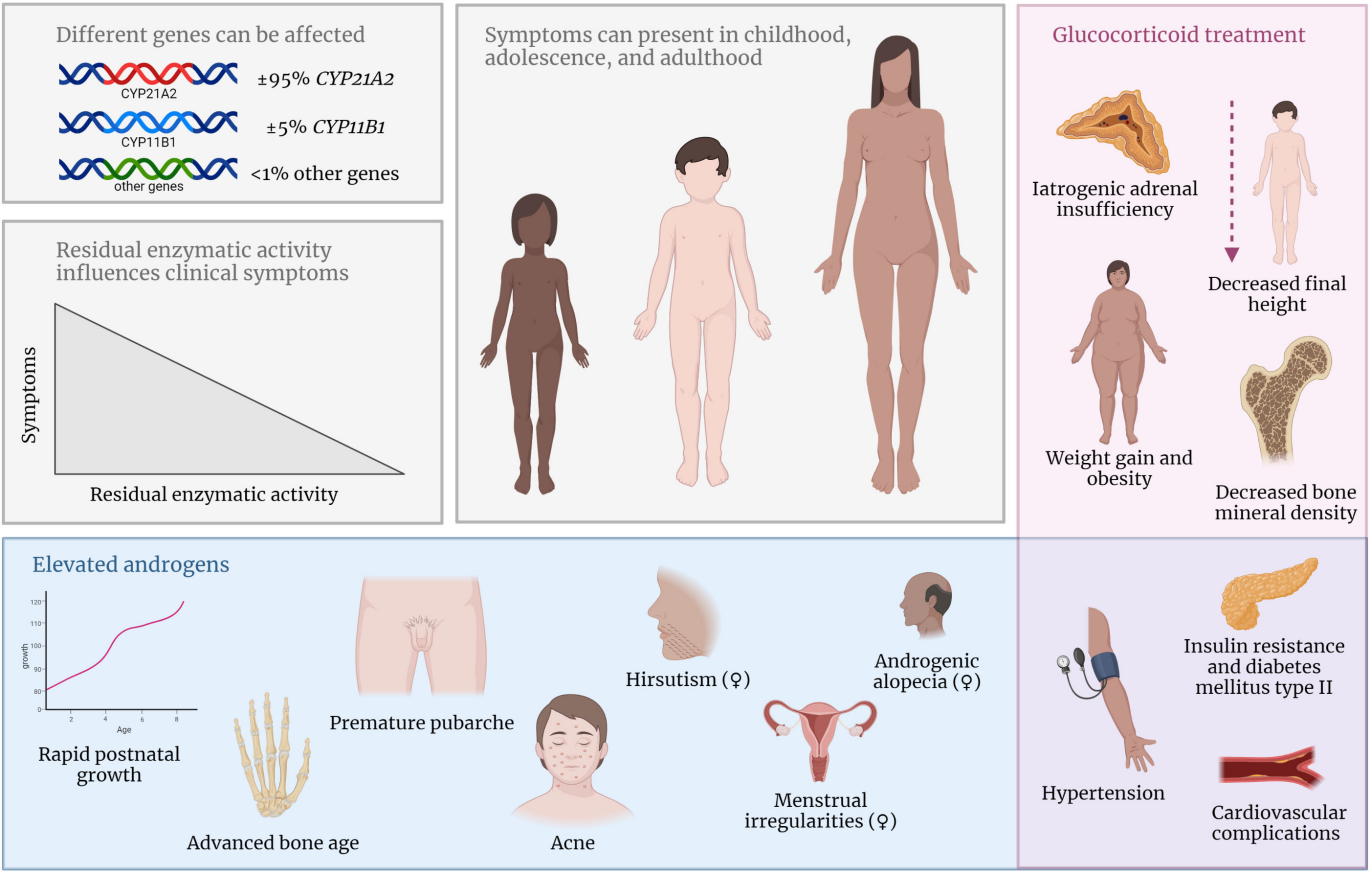
In NCCAH, different gene mutations can lead to decreased activity of the effected enzyme. Symptoms can present during childhood, adolescence, and adulthood and are influenced by the residual enzymatic activity. Elevated androgen concentrations can lead to various clinical symptoms (blue box). Side effects of supraphysiological glucocorticoid treatment are common (red box). Symptoms in the purple box occur due to elevated androgens or glucocorticoid treatment.

The severity of the disease and clinical presentation of 21OHD depends on the residual enzymatic activity (**Figure 2**) (4). Although the clinical spectrum is a gradual scale, 21OHD is historically classified into three groups. In the classic salt-wasting (SW) form, there is a (near) complete loss of enzymatic activity (< 1%) leading to a complete deficiency of both cortisol and aldosterone. The aldosterone deficiency in SW patients leads to neonatal salt loss which might be fatal if not recognized and treated. A residual enzymatic activity of 1 – 2%, referred to as the classic simple virilizing (SV) form, is needed to produce sufficient aldosterone and prevent salt wasting (3). Patients with a residual 21α-hydroxylase activity of 20-50% have a less severe phenotype and are grouped as non-classic CAH (NCCAH) (5).

In many countries, patients with classic 21OHD are diagnosed early in life by neonatal screening programs incorporating the measurement of 17-OHP concentrations in dried blood spots. However, in NCCAH patients, basal 17-OHP levels are generally not markedly elevated. CAH can also be diagnosed by the quantification of elevated adrenal steroids in serum taken before and after ACTH stimulation. In addition, genotyping can be performed to confirm the diagnosis in suspected cases.

There is generally sufficient cortisol and aldosterone production during basal conditions, although suboptimal cortisol levels have been described in some NCCAH patients after a stimulation test (6–9). The estimated prevalence of NCCAH is 1 in 200-1,000 in the Caucasian population (10, 11) with a higher prevalence in specific ethnic groups such as Ashkenazi Jews (12).

Due to the enzyme deficiency in both classic CAH and NCCAH patients, the precursor steroids before the enzymatic block increase and are partially shunted into the unaffected adrenal androgen synthesis pathway leading to a variable degree of increased adrenal androgen levels. In the SW and SV form, prenatal androgen concentrations are higher possibly leading to virilization of the external genitals in girls, already *in utero*. In NCCAH patients, androgen levels are lower compared to classic patients, and symptoms of hyperandrogenism present later in life. NCCAH patients may present with mild symptoms such as premature pubarche, accelerated bone age during childhood, acne in both boys and girls, and irregular menstruation and/or hirsutism in women (**Figure 2**). However, many patients are asymptomatic, especially adult males, because testicular androgen production of adult males surpasses adrenal androgen production (13). The clinical phenotype of female NCCAH patients might resemble the phenotype of women with polycystic ovarian syndrome, therefore, it can be challenging to distinguish between these diseases (14).

There is a gradual scale in 21OHD phenotypes and there are no specific cut-off values between classic and non-classic 21OHD. In general, treatment guidelines for classic CAH patients recommend glucocorticoid treatment to substitute for low serum cortisol concentrations and to suppress elevated ACTH to lower adrenal androgen synthesis. However, for NCCAH patients, general guidelines for monitoring and treatment are scarce, and the evidence for recommendations in guidelines is low (11, 15–17).

In this review, we will discuss the clinical aspects of NCCAH patients in childhood, adolescence and adulthood. We will first focus on patients with 21OHD and discuss the effect of treatment on different clinical outcome measures. Thereafter, we will shortly discuss patients with 11-hydroxylase deficiency (11OHD).

## Cortisol production in NCCAH

### Basal cortisol production

In contrast to classic 21OHD patients in which cortisol production is significantly impaired, most patients with non-classic 21OHD have basal cortisol levels within normal reference ranges when measured by immunoassay (7–9, 18). It should, however, be noticed that immunoassays are not free of cross-reactivity to precursor steroids that are generally increased in (NC)CAH patients leading to a possible overestimation of the cortisol concentration. Therefore, liquid chromatography tandem mass spectrometry (LC-MS/MS) should be used, which is currently the gold standard for quantifying steroid hormones, especially in patients with defects in the steroidogenesis (11).

Oriolo et al. (18) measured cortisol in NCCAH patients by LC-MS/MS in fasting serum taken between 8.00 and 8.30 am. Interestingly, they reported mean (± SD) cortisol levels (383 nmol/L ± 183) that were not significantly different compared to heterozygous carriers of 21α-hydroxylase mutations or women with polycystic ovarian syndrome. In addition, Ueland et al. (19) reported normal cortisol levels (median 346 nmol/L, range 140 – 771) in their cohort of NCCAH patients and heterozygous carriers. Even the lowest reported cortisol level (140 nmol/L) in this cohort of eight patients was within the range of the control group (108 – 653 nmol/L) (20). The absence of biochemical cortisol deficiency is supported by two studies that reported normal ACTH levels in NCCAH patients (7, 21), indicating sufficient negative feedback from cortisol on the pituitary gland.

These findings are in line with the clinical observations that manifestations of cortisol deficiency are uncommon in untreated NCCAH patients (7, 8). Stoupa et al. (8) reported that 33 out of 35 NCCAH patients (94.3%) showed no signs of hypocortisolism under basal conditions. The remaining two patients (5.7%) suffered from fatigue. These two patients were sisters, and both carried the same genetic defects, namely a V281L mutation and a large gene conversion, from which the latter is a severe mutation.

As most NCCAH patients do not have decreased cortisol production, suppletion with glucocorticoids is not recommended and might even be potentially harmful as glucocorticoids will suppress the pituitary-adrenal axis with consequently the need to use stress dosing in situations of illness. El-Maouche et al. (22) reported Addisonian crises in NCCAH patients receiving hydrocortisone treatment. In addition, Oliveira et al. (23) reported that 15% of their treated NCCAH patients did have at least one episode of acute adrenal insufficiency, while none of their untreated NCCAH patients did.

In conclusion, NCCAH patients have normal basal cortisol production. General treatment with glucocorticoids is not recommended and may even lead to iatrogenic adrenal insufficiency with a risk of developing an Addisonian crisis (**Figure 2**).

### Cortisol production during periods of physical stress

During physical stress, such as sick days or surgery, ACTH levels increase leading to increased production of cortisol, which is necessary to modulate the immune response and maintain adequate blood pressure (24, 25).

An ACTH stimulation test, in which cortisol and other adrenal steroids are measured after ACTH administration, is currently the gold standard to assess cortisol production in a clinical setting. A suboptimal cortisol response is reported in about 21-60% of NCCAH patients (6–9, 26). Nandagopal et al. (7) and Stoupa et al. (8) reported no significant correlation between the cortisol response to synthetic ACTH and the genotype of the patients.

Nonetheless, symptoms of acute adrenal insufficiency during periods of illness are seldomly reported in untreated NCCAH patients (6–9). Bidet et al. (6) described a cohort of 161 untreated NCCAH women (age range 13 – 52 years old) of whom two (1.2%) experienced signs of acute adrenal insufficiency. One patient had high fever for several days due to pyelonephritis and the other patient suffered from repeated vomiting in the first trimester of pregnancy. Both patients survived without the administration of hydrocortisone treatment. Nandagopal et al. (7) described three out of eight NCCAH patients (aged 27, 49, and 66) with a suboptimal cortisol response after ACTH stimulation but without signs of acute adrenal insufficiency during physical stress in their life, even though two of them underwent surgeries, such as Cesarean section, cholecystectomy, and bilateral ankle surgery. In addition, Karachaliou et al. (9) described a cohort of 31 pediatric NCCAH patients of whom seven (21.2%) had impaired cortisol levels after ACTH stimulation. None of these patients experienced signs of acute adrenal insufficiency during their life. These authors question the necessity of stress dosing in NCCAH patients, even in periods of severe stress. However, large cohort studies are necessary to confirm this.

The lack of symptoms, even in patients with a suboptimal cortisol response after ACTH, might be explained by several hypothetical mechanisms:

Firstly, several other steroid hormones are able to bind and activate the glucocorticoid receptor. The ligand binding domain of the glucocorticoid and mineralocorticoid receptors are structurally very similar and cross-reactivity of different adrenal steroids has been demonstrated before (27). Actually, corticosterone, an intermediate in the aldosterone synthesis pathway, is known to have glucocorticoid effects (28) and is the most important glucocorticoid in birds (29) and rodents (30). Specific hydroxyl groups at positions 11, 17, and 21 enhance binding affinity with the glucocorticoid receptor and subsequent glucocorticoid activity (31). Cortisol has a hydroxyl group at these three positions and is known as the endogenous steroid hormone with the greatest affinity for the glucocorticoid receptor in humans. However, other steroids with only two out of three hydroxyl groups can also have a glucocorticoid effect. In 21OHD, 17-OHP and 21-deoxycortisol are elevated (**Figure 1**). This latter steroid hormone has a hydroxyl group at positions 11 and 17 and Engels et al. (32) reported that 21-deoxycortisol can bind, translocate, and activate the glucocorticoid receptor *in vitro* with a relative potency of 49% compared to cortisol. In addition, 21-deoxycorticosterone, an 11-hydroxylated progesterone molecule, can activate the receptor with a relative potency of 23% compared to cortisol. During periods of physical stress, ACTH increases to stimulate the adrenal cortex. Due to the enzyme deficiency, steroids prior to the enzymatic block, such as 21-deoxycortisol (33) and 21-deoxycorticosterone (34), increase. These precursor steroids may partially compensate for the insufficient cortisol response found in some NCCAH patients and thereby prevent signs of cortisol deficiency.

Secondly, the total cortisol concentration that is generally measured might give an inaccurate reflection of the biological glucocorticoid activity. The total cortisol concentration consists of the cortisol bound to proteins ( ± 90%), mostly corticosteroid binding globulin, and unbound (i.e., free) cortisol. Only free cortisol activates the glucocorticoid receptor (35–39). It has been reported that other steroid hormones, that are increased in 21OHD patients, such as testosterone, progesterone, 17-OHP, and 21-deoxycortisol, can influence the binding of cortisol to corticosteroid binding globulin (40–44), possibly increasing free cortisol levels in NCCAH patients.

Lastly, polymorphisms in the glucocorticoid receptor have been associated with differences in sensitivity (45), possibly increasing the sensitivity in some NCCAH patients. Further research is necessary to better understand individual differences in glucocorticoid sensitivity and how we can implement this in routine clinical CAH care.

In conclusion, a suboptimal cortisol response after ACTH stimulation is reported in up to 60% of untreated NCCAH but symptoms of an Addison crisis are not often reported. Therefore, current guidelines recommend stress dosing in those patients with a suboptimal ACTH test only in severe stress situations.

## Growth

Untreated and poorly treated children with classic 21OHD show accelerated growth, advanced bone age with early epiphyseal fusion, and subsequently reduced final height due to overproduction of adrenal androgens (46, 47). The effects of adrenal androgens on (increased) bone maturation become already relevant in the second year of life (48, 49). On the long term, these androgens lead to premature epiphyseal closure resulting in reduced final height.

In contrast, in untreated NCCAH patients, reported final heights are within the normal range in most studies (6, 50–52), suggesting that the elevated androgens in these patients do not lead to significant growth reduction. However, one study by New et al. (53) reported a final height below the target height in untreated NCCAH patients. Mutation analyses were not performed in this study, so it is unclear whether differences in genotype could explain the discrepancy between the studies. Einaudi et al. (54) reported that untreated pediatric NCCAH patients with one of the two affected *CYP21A2* mutations classified as moderate or severe (e.g., Q318X, IVS2, or R356W) showed a higher height SDS compared to patients with two mutations classified as mild (e.g., V281L, P453S, or P30L). This is probably due to the higher levels of androgens in the former group leading to growth acceleration during childhood (**Figure 2**).

To avoid the unwanted effects of adrenal androgens on growth, children with CAH are treated with glucocorticoids to restore the negative feedback on the pituitary gland and consequently decrease the overproduction of adrenal androgens. Mostly, supraphysiological dosages of glucocorticoids are necessary to reach this goal (55), especially during puberty, because cortisol pharmacokinetics changes in this period resulting in increased cortisol clearance (56). The estimated endogenous cortisol production is 5.3 – 7.4 mg/m^2^/day (57–60), while recommended dosages for pediatric CAH patients are 10 – 15 mg/m^2^/day. Unfortunately, supraphysiological glucocorticoid dosages may suppress growth in children as well and cause additional weight gain in children and adults (**Figure 2**), especially when long-acting glucocorticoids like prednisone or dexamethasone are used (61–64). Therefore, finding a balance between over- and undertreatment in CAH patients is often challenging and the prescription of long-acting glucocorticoids should be avoided in children (65).

There is no clear evidence that final height is significantly decreased in NCCAH patients receiving glucocorticoid treatment during childhood (47, 51, 66). Wasniewska et al. (51) reported no significant difference in final height between treated and untreated patients. In contrast, Eyal et al. (52) reported reduced final height in NCCAH patients after receiving hydrocortisone compared to untreated patients. However, the group that received treatment during childhood was diagnosed earlier than the untreated group. In addition, there was a significant difference in genotype between the two groups, the occurrence of two mild mutations was respectively 70% in the treated group versus 89% in the untreated group. Furthermore, also heterozygous carriers were included in this study, possibly causing an underestimation of the effect of elevated androgens and glucocorticoids on final height. Weintrob et al. (67) found that the age of initiation of glucocorticoid treatment may also influence final height: NCCAH patients in whom treatment was started at least one year before the onset of puberty had a better height outcome compared to patients who started treatment after the onset of puberty. However, in the group of patients who started treatment after the first signs of puberty, three out of eight had precocious puberty, which might result in compromised final height and may have influenced the results of this study.

Thus, phenotypic heterogeneity, as well as differences in patient population and treatment regimens makes the interpretation and comparison of the results less reliable.

Clinically, growth acceleration is often small in untreated children with NCCAH (68). Bone age can be used as an additional clinical parameter besides monitoring growth velocity to evaluate the effect of adrenal androgens on growth (54, 68). A progressive bone age acceleration may be an indication to start glucocorticoid treatment, but careful counseling about the advantages and disadvantages of this treatment should be offered to patients and parents including the need to use stress dosing. Glucocorticoid treatment can be discontinued when final height is reached. Discontinuation of glucocorticoid treatment in NCCAH patients decreases the risk of long-term complications such as iatrogenic Cushing syndrome with excessive weight gain (69).

In conclusion, negative effects of adrenal androgens in untreated pediatric NCCAH patients are generally mild but patients with more advanced bone age acceleration are described. Therefore, yearly follow-up of growth and bone age is recommended. Glucocorticoid treatment should only be initiated in children with NCCAH after careful counseling of patients and parents and should be discontinued after reaching the final height.

## Puberty

Premature pubarche, defined as the presence of pubic hair before the age of 8 years in girls and 9 years in boys (70), is the most common symptom of androgen excess in prepubertal children with NCCAH with an incidence of 55-92% (71–73). Chronically elevated adrenal androgens can also increase the GnRH pulse frequency in GnRH neurons (74) and can, thereby, potentially activate the pituitary-gonadal axis leading to earlier onset of puberty (71, 75–77). Puberty onset in NCCAH patients is earlier compared to the average population (47, 67) but, in general, within the physiological range. The age of puberty onset is related to the genotype; compound heterozygous patients with one severe mutation (e.g., Q318X, I2 splice, or I172N) and one mild mutation (either V281L or P30L) tend to have an earlier onset of puberty compared to patients with two mild mutations (78). However, true central precocious puberty, defined as activation of the hypothalamus pituitary gonadal axis leading to breast development before the age of 8 years in girls or a testicular volume ≥4 mL before the age of 9 years in boys, is only seen in about 4-5% of untreated NCCAH patients (71). Therefore, hydrocortisone treatment is not recommended to prevent central precocious puberty.

In boys, a testes volume of ≥4 ml indicates an activation of the gonadal axis with increased production of gonadal testosterone (79). During puberty, testicular androgen production greatly overshoots the adrenal androgens production in boys and enduring suppression of androgens seems unfavorable. Therefore, Merke et al. (69) recommend discontinuation of glucocorticoid treatment at a testicular volume of 8-10 mL (Tanner stage 3) when glucocorticoid treatment is used to prevent early pubertal development.

In NCCAH girls treated with glucocorticoids, the age of onset of puberty and menarche is reported as normal (23, 47, 54, 66, 67). One recent study (23) reported a significantly earlier age of menarche in NCCAH patients compared to classic patients but results are hard to compare as all classic patients were treated with glucocorticoids, while 13% of the NCCAH patients were untreated. However, the median age of menarche was still in the normal range in both classic and NCCAH patients. Einaudi et al. (54) described a correlation between age of menarche and severity of the mutation between subtypes of NCCAH, but the median age of menarche was within the normal range for all subtypes. Approximately half of the female NCCAH patients suffer from oligomenorrhoea (72) due to increased production of adrenal androgens, which are aromatized to estrogens, and elevated adrenal progesterone production, both leading to suppression of the hypothalamic-pituitary-gonadal axis (80). Menstrual regularity can be achieved by oral contraceptives with antiandrogenic effects (81).

In conclusion, premature pubarche is a common symptom in NCCAH patients but central precocious puberty is rare and hydrocortisone treatment to prevent precocious puberty is not recommended. If central precocious puberty occurs, hydrocortisone treatment can be started to improve final height, but only after careful counseling.

## Transition into adult care

Patients with NCCAH in adolescence and adulthood have reached their final height and completed their pubertal development. Therefore, other treatment goals are important during these stages. Before transition into adult care it is important to assure regular monitoring, education about the disease and long-term follow up (82). Therewith, patients have sufficient information about the disease and the treatment to be self-dependent if necessary. In the next paragraphs, we will discuss the different clinical parameters that should be considered during adolescence and adulthood.

## Bone health

Low (83–98), normal (99–105), and high (106) bone mineral densities (BMD) are reported in studies in which both classic and NCCAH patients were included. However, knowledge about bone health in NCCAH patients is scarce because most studies do not present data of NCCAH patients separately (84, 92, 93, 96, 97, 102). It is known that glucocorticoid treatment can potentially affect bone health. If NCCAH patients use glucocorticoids, dosages are usually comparable to the dosages used in classic patients (55), so the long-term side effects of glucocorticoid treatment are most likely similar. However, in NCCAH, glucocorticoid treatment is most often initiated at a later age, and before that time NCCAH patients are exposed to androgens which is favorable for BMD (see below). This is also illustrated in several studies where higher bone quality or BMD is reported in NCCAH patients compared to classic CAH patients (85, 89, 105, 107, 108). Finkielstain et al. (94) reported comparable BMD between classic and NCCAH patients. In this same study, older age was associated with lower femoral neck BMD in classic CAH patients but not in NCCAH patients. This might indicate that prolonged glucocorticoid treatment has a negative effect on femoral BMD in classic patients but less in NCCAH patients (94).

The inconsistency between studies may be due to differences in the patients’ age of BMD determination. Besides, several factors play a role in the bone health of NCCAH patients:

Firstly, androgens stimulate osteoblast proliferation and differentiation and inhibit osteoclast formation (109), thereby, increasing BMD. This was illustrated by one study reporting higher lumbar spine BMD SDS in classic CAH prepubertal patients who did only receive glucocorticoid treatment for a relatively short period (106).

Secondly, glucocorticoids have direct effects on bone metabolism. They increase bone resorption by upregulation of osteoclasts (110) and increase their life span (111). In addition, glucocorticoids directly induce osteoblast and osteocyte apoptosis which subsequently leads to decreased bone formation (112). Furthermore, osteoblasts produce fewer vascular growth factors during glucocorticoid treatment which inhibits bone vascularization leading to bone necrosis (113). These glucocorticoid effects have a negative impact on bone formation, especially on trabecular bone (114) which is mainly present in the vertebral bodies and epiphyses of long bones (115).

Thirdly, glucocorticoids have an indirect effect on BMD by inhibiting calcium absorption in the gastrointestinal tract and calcium reabsorption in the renal tubules (116).

Lastly, glucocorticoids decrease the concentration of androgens (117), thereby attenuating the positive effect of the increased androgen concentration on BMD in NCCAH patients.

It is generally known that glucocorticoids have potential negative effects on BMD, also observed in patients who use glucocorticoids for various reasons (114). Long-acting glucocorticoids like dexamethasone and prednisolone have a less favorable effect on BMD compared to short-acting glucocorticoids like hydrocortisone (84, 118).

In general, the use of glucocorticoid treatment increases the risk of fractures (119). A recent study from Falhammar et al. (120) reported an increased frequency of fractures in classic CAH patients but not in NCCAH patients. This is consistent with the findings of another study that reported fewer non-traumatic fractures in NCCAH patients compared to CAH patients (108). However, when only major osteoporotic fractures are considered, both forms of CAH had an increased frequency ( ± 5% in NCCAH vs ±10% in classic CAH patients) compared to the general population (120). This indicates that glucocorticoid treatment has clinically relevant negative effects on bone health. When glucocorticoid use is discontinued, the fracture risk decreases (114, 119), indicating that the glucocorticoid effects are reversible. If patients are untreated, no routine dual-energy X-ray absorptiometry is necessary to evaluate BMD. The Endocrine Society suggests screening for BMD only in patients receiving high dosages of glucocorticoids or who suffered from a non-traumatic fracture (11).

Besides the use of glucocorticoids, other risk factors like smoking, low intake of calcium and vitamin D, and low physical activity increase the risk of osteoporotic fractures (121). It is essential to inform NCCAH patients about these risk factors to preserve their bone mineral density and reduce the risk of osteoporotic fractures later in life.

In conclusion, treatment with glucocorticoids during childhood can already negatively influence BMD later in life. Therefore, both endocrinologists and pediatric endocrinologists should include these (long-term) negative effects in the decision whether glucocorticoid treatment is beneficial for their particular patient and to properly counsel their patients. When glucocorticoids are necessary, hydrocortisone should be used, especially in children, as this short-acting glucocorticoid has fewer negative effects on BMD compared to long-acting glucocorticoids. Patients should be counseled for the potential decrease in BMD, and other risk factors for osteoporotic fractures like smoking, low calcium and vitamin D levels, and low physical activity should be brought to a minimum.

## Cardiovascular and metabolic complications

NCCAH is associated with increased cardiovascular and metabolic morbidity in adulthood (122). Both glucocorticoid treatment and androgen excess are important factors in this respect (**Figure 2**). Supraphysiological glucocorticoid dosages are associated with a higher prevalence of obesity, insulin resistance, dyslipidemia, and hypertension, which are known risk factors for cardiovascular disease (123, 124). On the other hand, untreated or undertreated 21OHD patients suffer from androgen excess which can also be unfavorable for the cardiovascular and metabolic risk profile (125). In this paragraph, different cardiovascular and metabolic complications and risk factors in NCCAH patients will be discussed.

Several studies (92, 94, 122) reported a higher prevalence of obesity in glucocorticoid-treated NCCAH patients compared to healthy controls. Falhammar et al. (126) reported a higher BMI and waist-to-hip ratio in treated NCCAH women (compared to controls) who were ≥30 years of age, but not in patients <30 years. This indicates that the effect on body composition and fat distribution occurs later in life. Several factors may cause weight gain in CAH patients (127). Völkl et al. (128) reported a minor positive correlation (r=0.22, p=0.04) between hydrocortisone dose and BMI, suggesting that supraphysiological dosages of glucocorticoids contribute to weight gain. Ariyawatkul et al. (129) did, however, not confirm this finding, possibly because a smaller sample size was used. Zhang et al. (130) reported a higher BMI in 30 untreated female patients with classic simple virilizing 21OHD, indicating that glucocorticoids are not the only factor influencing the weight gain in classic 21OHD and that the elevated androgens are possibly a contributing factor as well. These authors suggested that the elevated androgens have a negative effect on the body fat distribution and lipid metabolism. Nonetheless, Saygili et al. (125) reported no significant difference in BMI between untreated NCCAH patients and healthy controls even though free testosterone levels were four times higher in the patient group compared to the controls. The difference between the two studies might be explained by the fact that the former study included more severe cases of 21OHD compared to the latter (simple virilizing vs. NCCAH) and the included patients had higher concentrations of androgens. This suggests that slightly increased androgen levels in NCCAH do not affect BMI.

Another factor influencing cardiovascular and metabolic risk is insulin sensitivity. Several studies reported increased insulin resistance in both treated and untreated NCCAH patients compared to controls (90, 92, 125, 131–133). Williams et al. (132) confirmed this finding in treated NCCAH patients but did not report a higher prevalence of insulin resistance in classic CAH patients. In this study, classic CAH patients were diagnosed by newborn screening and subsequently treated. The NCCAH patients were diagnosed at a later age and, therefore, exposed to elevated androgen concentrations for a longer postnatal period. This suggests that hyperandrogenism contributes to insulin resistance in these NCCAH patients. This is confirmed by other studies (14, 134, 135) who reported higher rates on insulin resistance in women with polycystic ovarian syndrome, who also suffer from hyperandrogenism. There is a vicious circle in which hyperandrogenism leads to insulin resistance, resulting in hyperinsulinemia, which in turn leads to an aggravation of hyperandrogenism (135). On the contrary, Bayraktar et al. (136) did not report increased insulin resistance in NCCAH patients compared to controls. As genetic testing was not performed here, differences in disease severity of these patients versus patients included in other studies could not be evaluated. Besides hyperandrogenism, also glucocorticoids lead to insulin resistance by opposing the actions of insulin (137). Delai et al. (133) confirmed this in NCCAH patients and found that insulin resistance in patients was related to prolonged use of long-acting glucocorticoids.

Whether the increased insulin resistance also leads to a higher incidence of diabetes mellitus type II was unknown for a long time, because only a few patients were older than 50 years old in the described studies and diabetes usually presents later in life. Falhammar et al. (138) did not report higher incidences of diabetes in one study but sample sizes were small and the oldest included patient was 67 years old. In a later study, Falhammar et al. (122) found a higher prevalence of diabetes in a cohort of 75 treated NCCAH patients (with the oldest patient being 92 years old) compared to age- and sex-matched controls.

Another important risk factor for cardiovascular diseases is hypercholesterolemia. Hypercholesterolemia has been observed in 59% of the NCCAH females (92). This incidence was even higher compared to the classic CAH males and females (36% and 48% respectively) (92). Krysiak et al. (139) reported that atorvastatin, a statin that reduced the levels of cholesterol and androgens, decreased the cardiometabolic risk in untreated NCCAH women. If this decrease in risk also leads to fewer cardiovascular incidents in these patients, needs to be further elucidated.

In conclusion, glucocorticoid treatment has potential negative effects on the cardiovascular and metabolic system (123, 124). These complications are dose-dependent (123, 140) and, therefore, glucocorticoids should always be prescribed in the lowest effective dose (124). However, hyperandrogenism also contributes to an unfavorable cardiometabolic risk profile, and therefore adequate monitoring and balancing over- and undertreatment is necessary. Other treatment options like statins need further attention, to elucidate whether these can be used in untreated NCCAH patients with hyperandrogenism to reduce the risk for cardiometabolic complications.

## Dermatological symptoms caused by hyperandrogenism

Hyperandrogenism can lead to well-known clinical symptoms such as hirsutism, acne vulgaris, and androgenetic alopecia (**Figure 2**). In NCCAH, these symptoms of androgens excess can present already during adolescence but are mostly observed later in life. Hirsutism is the most common symptom in adult women with NCCAH and does not correlate well with genotype (54). In childhood, hirsutism is only observed in 4% of the patients, while in adulthood the incidence rate increases to 69-78% (6, 72). Alopecia incidence rates also increase with age with peak incidences of 19% between 40-49 years of age (72). Acne is most often observed in 20- to 29-year-olds with an incidence of 37% (72). Signs of adrenal hyperandrogenism are less frequently observed in men, as testicular androgen production greatly outreaches adrenal androgen production (13).

Glucocorticoids can be effective in lowering adrenal androgen levels and thereby also decreasing the dermatological signs of hyperandrogenism. However, due to long-term negative effects on bone health and cardiovascular risk as discussed above, other drugs like oral contraceptives with antiandrogenic effects are generally used as first step treatment option in females with NCCAH suffering from hirsutism. Different mechanisms contribute to the antiandrogenic effect of oral contraceptives. First, estrogens have negative feedback on the pituitary gland, thereby lowering the levels of luteinizing hormone resulting in less ovarian androgen production in females and testicular androgen production in males (141). In addition, oral contraceptives lower adrenal androgen production by inhibiting the enzymatic activities of 17-hydroxylase and 17,20-lyase which are necessary for androgen production (142). Furthermore, estrogens in oral contraceptives increase sex hormone binding globulin (SHBG) production and thereby reduce free testosterone levels (143, 144). Lastly, progestogens in oral contraceptives inhibit the function of 5α-reductase resulting in less conversion of testosterone into the more potent androgen dihydrotestosterone (145). Although oral contraceptives can contain different progestogens with either androgenic or antiandrogenic features, the net effect of combined oral contraceptives is always antiandrogenic (146). Antiandrogenic progestogens can also be used as monotherapy. For instance, cyproterone acetate monotherapy has been described to be superior compared to hydrocortisone in treating hirsutism in female NCCAH patients (147).

Another option interfering with the androgen pathway to reduce signs of hyperandrogenism is the administration of spironolactone. This mineralocorticoid receptor antagonist has antiandrogenic effects as it blocks the binding of (dihydro)testosterone to the androgen receptor and diminishes 5α-reductase activity in the skin (148, 149). Physicians used to be hesitant to prescribe spironolactone for hirsutism or acne treatment because carcinogenic features of spironolactone are described (3). However, this was only seen in animal models using very high dosages of spironolactone (150). Higher incidence rates for cancer were not found in humans using spironolactone (151–153) and nowadays, the use of spironolactone in the treatment of acne is increasing (154).

Other off-label treatment options for hyperandrogenism are competitive antagonists of the androgen receptor, like flutamide, and 5α-reductase inhibitors, like finasteride and dutasteride (146). However, these should not be used in childhood and in women who try to conceive or are currently pregnant. These antiandrogens can have side effects like decreased libido, headache, dizziness, and nausea (155–157). In addition, flutamide is potentially hepatotoxic and liver functions should be monitored during treatment (158).

Besides antiandrogens, also cosmetic and/or topical treatment options have to be considered to treat hirsutism in female NCCAH patients. This includes, among others, shaving, plucking, waxing, laser therapy, or topical eflornithine cream (159, 160). For acne, topical gels with benzoyl peroxide, antibiotics, retinoids, or azelaic acid can be effective (161). A combination of different treatment approaches (both topical and systemic hormonal) might be most effective (160, 162).

In conclusion, NCCAH women may suffer from signs of hyperandrogenism such as hirsutism, acne vulgaris, or androgenetic alopecia. Glucocorticoid treatment is not recommended to reduce signs of hyperandrogenism in these patients. An individualized approach with topical and/or hormonal treatment is necessary for treating signs of hyperandrogenism, and consultation with a dermatologist can be of added value for these patients.

## Fertility and pregnancy in women

Oligomenorrhoea in NCCAH women may lead to sub- or infertility. However, reported pregnancy rates are normal in these women (163–166). Bidet et al. (167) reported that 57% of the pregnancies were spontaneous without any treatment and 83% of the women who conceived were pregnant within one year. Untreated mothers with 21OHD have elevated androgen levels but these androgens cannot reach the fetus because placental aromatase will convert them into estrogens protecting the female fetus from virilization (168).

If pregnancy is not achieved, temporary glucocorticoid treatment might be indicated to normalize progesterone levels (11, 167, 169). Here, hydrocortisone, prednisolone, or prednisone should be used as they can be inactivated by placental 11β-hydroxysteroid dehydrogenase type 2 and do not reach the fetus (170). Hydrocortisone or prednisone before and/or during pregnancy did not significantly change the duration of pregnancy or the child’s birth weight (165, 167). Placental 11β-hydroxysteroid dehydrogenase type 2 is not able to metabolize dexamethasone and dexamethasone will reach the fetus (171) which can lead to negative side effects (172). Therefore, we recommend against the use of dexamethasone in women who try to conceive or are already pregnant. If glucocorticoid treatment is necessary, an endocrinologist specialized in CAH should be involved.

Although pregnancy rates in NCCAH women are similar to those in the general population (163–165), these women have higher rates of miscarriages compared to healthy females (165–167, 173). This might be due to dysfunction of the corpus luteum in NCCAH women (174). The progesterone production of the corpus luteum is most important for the continuation of pregnancy in the first trimester (175) and this is also the period in which most miscarriages occur (165, 167).

Moran et al. (173) showed that miscarriage rates were lower in NCCAH women who were diagnosed before conception compared to NCCAH women who were diagnosed thereafter. The exact reason for this is unclear, but it is noteworthy that almost 65% of the women in the diagnosed group were treated with glucocorticoids (either alone or in combination with clomiphene or menotropins), compared to 5% of the women in the undiagnosed group. However, this study found no significant difference in miscarriage rate between untreated women compared to women treated with glucocorticoids alone (i.e., without clomiphene and/or menotropins). Whether these treated women received glucocorticoid treatment *before* or *during* pregnancy did not influence the miscarriage rate. Moreover, also Eyal et al. (165) did not observe a difference in miscarriage rate in treated versus untreated women. On the contrary, studies by Feldman et al. (166) and Bidet et al. (167) reported normalized miscarriage rates in NCCAH women with glucocorticoid treatment. This difference might be explained by the genotype of the patients; Bidet et al. (167) described patients with a more severe genotype (but both NCCAH) compared to Eyal et al. (165), which possibly results in a greater effect of the glucocorticoids. The other two studies (166, 173) did not report mutation analyses of the NCCAH women. If glucocorticoid treatment does not lead to conception, ovulation induction with clomiphene citrate might be successful (166, 167).

As CAH is a recessive disorder, NCCAH patients have two affected alleles. Therefore, the child has at least one mutated allele from the affected parent. If the partner is heterozygous for 21OHD or a *de novo* mutation occurs, the child can suffer from CAH with also the risk of a child with a classic CAH. Higher incidence rates of both classic and NCCAH were found in children of NCCAH mothers (165, 167, 173). Therefore, genetic counseling is recommended to inform parents about this risk and to genetically test the partners of patients with NCCAH (167).

In conclusion, reported pregnancy rates are normal in NCCAH women. Therefore, glucocorticoid treatment is generally not advised. There is no conclusive evidence whether glucocorticoid treatment during pregnancy in female NCCAH patients leads to a decreased miscarriage rate. It is recommended that an endocrinologist specialized in CAH is involved before and during pregnancy of NCCAH women. If glucocorticoid treatment is indicated, we advise prescribing hydrocortisone to prevent glucocorticoids to cross the placental barrier.

## TART and fertility in men

In classic CAH, benign testicular adrenal rest tumors (TART) can lead to decreased fertility in men (176–179). Four different articles (94, 98, 180, 181) reported the incidence of TART in NCCAH men and described a total of 124 NCCAH patients of whom only two had evidence of TART. So, TART are not a common finding in NCCAH males, and fertility in men with NCCAH is generally normal. Therefore, routine ultrasound of the scrotum is not recommended in NCCAH men (3).

## 11OHD

The second most common form of CAH is 11OHD with a diminished conversion of 11-deoxycortisol into cortisol (**Figure 1**) (182). The estimated prevalence of 11OHD in the general population is 1 in 100,000 (183). As the clinical picture of NC 11OHD is variable and could resemble that of women with polycystic ovarian syndrome, its prevalence is most likely underestimated. Interestingly, a prospective study identified no NC 11OHD patients amongst 270 women with hyperandrogenism, whilst six suffered from NC 21OHD (184), indicating that the prevalence of 11OHD is probably lower than the prevalence of NC 21OHD.

In non-classic 11OHD, residual enzymatic activities ranging from 15 to 73% are reported (185, 186). Steroid hormones prior to the enzymatic block, such as 11-deoxycortisol, progesterone, 11-deoxycorticosterone, and 17-OHP increase, of which the latter is converted into adrenal androgens by the unaffected adrenal androgen pathway (**Figure 1**). Therefore, the non-classic form of 11OHD might also present with signs of hyperandrogenism like precocious pseudo puberty in children or irregular menstruation in females, similar to NC 21OHD patients (187, 188). Treatment management of 11OHD patients also resembles the treatment in 21OHD patients.

Classic 11OHD patients have elevated 11-deoxycorticosterone levels which can bind the mineralocorticoid receptor resulting in high blood pressure, low renin levels, and hypokalemia (189). As 11-deoxycorticosterone prevents salt wasting from happening, no salt-wasting form of 11OHD is known. Salt wasting was only observed after glucocorticoid treatment, as this gives negative feedback on the pituitary gland and, thereby, decreases the ACTH concentration, eventually leading to reduction of the 11-deoxycorticosterone concentration (190). Most NC 11OHD patients do not present with hypertension during childhood (187, 191). However, the occurrence of hypertension in some patients cannot be ruled out because residual enzymatic activity is variable and the clinical picture is a continuum as seen in 21OHD (187). In fact, hypertension is observed in some patients with a mild form of 11OHD (188, 191, 192) and Zachmann et al. (191) found a correlation between the age of diagnosis and systolic blood pressure. This indicates that hypertension can occur in the NC form as well, but reported incidence rates are lower compared to patients with classic 11OHD (191).

Glucocorticoid treatment will restore the negative feedback on the pituitary gland similar to treatment in patients with 21OHD. This might be useful in patients with classic 21OHD but potentially harmful in classic 11OHD patients. Mineralocorticoid activity in 11OHD patients relies on 11-deoxycorticosterone which is in these patients ACTH dependent (193). By administration of glucocorticoids, ACTH will decrease and less 11-deoxycorticosterone is produced, increasing the chance of salt wasting crisis in classic 11OHD patients (190). However, it is unclear to what extent this is relevant for NC 11OHD patients as residual activity of 11β-hydroxylase will probably secure sufficient aldosterone production. If hypertension is present, treatment with mineralocorticoid antagonists like spironolactone might be successful (188, 192). Similar to NC 21OHD patients, 11OHD patients who receive glucocorticoid treatment have an increased risk for an iatrogenic Addisonian crisis if adherence to glucocorticoid treatment is poor and the patient becomes sick (22).

As the clinical picture of 11OHD patients is variable and glucocorticoid treatment has both short- and long-term side effects, it should be considered per patient whether administration of glucocorticoids is beneficial.

## Discussion

In this review, we focus on different clinical aspects of NCCAH patients in childhood and adulthood. Most NCCAH patients have cortisol levels within the normal reference ranges and normal ACTH levels, indicating that there is sufficient glucocorticoid activity during basal conditions (i.e., in periods without physical stress). Hence, the main problem in NCCAH patients during basal conditions is not the decreased cortisol production, but the increased production of adrenal androgens.

Hyperandrogenism can lead among others to rapid postnatal growth, advanced bone age, and premature pubarche in childhood, as well as acne, hirsutism, menstrual irregularities (in females), and decreased insulin resistance in adulthood.

Although glucocorticoid treatment could suppress the production of ACTH in the pituitary gland and lower adrenal androgen production, most NCCAH patients do not suffer from clinically relevant glucocorticoid deficiency. Therefore, glucocorticoid treatment is generally not recommended in NCCAH. Glucocorticoid treatment can have a negative impact on the cardiovascular system, metabolic outcome, and bone health later in life. Besides, externally administered glucocorticoids can lead to iatrogenic adrenal insufficiency during periods of sickness with a risk of developing a life-threatening Addisonian crisis, whilst most untreated NCCAH patients do not develop this severe complication. Therefore, patients should be carefully evaluated and the decision for starting glucocorticoid treatment should be made for each patient individually taking into account whether treatment solely aimed to lower androgen levels outweighs the negative effects of chronic glucocorticoid treatment. Adequate counseling should be offered to patients (and their parents).

In **Table 1** we present general recommendations for NCCAH patients based on the literature discussed in this review. It should be noted that it is difficult to predict which NCCAH patients will develop symptoms of hyperandrogenism because the residual enzymatic activity is a gradual scale that leads to a variable degree of androgen excess in different patients. Furthermore, several genes can be variably affected by different mutations, leading to different residual activities of the affected enzyme. In addition, not all forms of NCCAH show a good genotype-phenotype correlation, and different factors contribute to the clinical presentation. Therefore, an individualized follow-up and treatment plan is of utmost importance and clinicians should always weigh which treatment options are best for their patient.

**Table 1:** Recommendations for monitoring and treatment of NCCAH patients.

Clinical parameter	Patient group	Recommendations
Daily glucocorticoid treatment	All ages	1. Daily glucocorticoid treatment is not recommended to compensate for a cortisol deficiency.
Stress dosing of glucocorticoids	All ages	1. Stress dosing is only recommended in patients with suboptimal ACTH test in periods of severe physiological stress such as major surgery, child delivery or trauma. 2. In NCCAH patients receiving basal glucocorticoid treatment stress dosing is recommended.
Growth	Childhood	1. Yearly follow up of growth and bone age until final height is reached. 2. Start glucocorticoid treatment in patients with severe accelerated bone age to improve final height only after careful counseling of patients and parents. 3. Use short-acting glucocorticoid like hydrocortisone, especially during childhood. 4. Discontinue glucocorticoid therapy when final height is reached.
Puberty	Childhood	1. Isolated premature pubarche is no indication for glucocorticoid treatment. 2. Glucocorticoid treatment is not advised to prevent central precocious puberty.
Menstruation (in girls en females)	Adolescence and adulthood	1. Daily glucocorticoid treatment is not recommended to regulate the menstrual cycle. 2. Antiandrogenic contraceptives are recommended as the first step in the treatment of menstrual irregularity.
Bone health	Adulthood	1. Screening for BMD is only recommended in NCCAH patients receiving supraphysiological dosages of glucocorticoids for a prolonged period 2. Patients using glucocorticoids should be informed about other risk factors for decreased bone mineral density such as smoking, low intake of calcium and vitamin D, and low physical activity.
Cardiovascular and metabolic system	Adulthood	1. Negative effects of glucocorticoids on the cardiovascular and metabolic system are dose-dependent and therefore glucocorticoids should always be prescribed in the lowest effective dose. 2. Both glucocorticoid treatment and elevated androgens (due to undertreatment) negatively influence the cardiovascular and metabolic system, but evidence in NCCAH is low
Dermatological signs of hyperandrogenism	Adolescence and adulthood	1. Daily glucocorticoid treatment is not recommended as a first step in the treatment of hyperandrogenism such as hirsutism, acne, or androgenic alopecia. 2. Treatment options to reduce hirsutism in adult female NCCAH patients: a. Topical treatment with eflornithine cream. b. Cosmetic treatment such as shaving, plucking, waxing, or laser therapy. c. Systemic treatment with spironolactone, competitive androgen receptor antagonists like flutamide or 5α-reductase inhibitors such as finasteride or dutasteride. d. A combination of these treatment options. 3. For acne, topical treatment with benzoyl peroxide, antibiotics, retinoids, or azelaic acid can be effective. If acne is severe, systemic treatment with antibiotics or retinoids can be used.
TART and fertility	Men in adolescence and adulthood	1. Routine ultrasound for TART screening is not recommended.
Fertility	Women in adulthood who try to conceive	1. Glucocorticoid treatment is only advised in females who do not get pregnant after a substantial period. 2. An endocrinologist specialized in CAH should be involved. 3. If glucocorticoid treatment is indicated, we advise to prescribe hydrocortisone because this glucocorticoid does not cross the placental barrier.
Pregnancy	Pregnant women	1. The use of dexamethasone during pregnancy is not recommended. 2. Genetic counseling is recommended to inform patients about the increased risk for classic and non-classic CAH in their offspring.

Further research is necessary to establish more evidence-based treatment recommendations specifically for NCCAH patients. Most studies described only classic 21OHD patients or cohorts of classic and NCCAH patients together. Findings in these patients cannot always be extrapolated to the situation of NCCAH patients. So, bias in the design of these studies may have resulted in misleading conclusions for NCCAH patients. Cohort studies in untreated NCCAH patients can be useful to gain more insight into the natural course of the disease. In addition, such studies will give more information about the risk of Addisonian crisis in untreated patients. Furthermore, more information is needed on differences in glucocorticoid sensitivity, glucocorticoid activity of adrenal precursor steroids, and differences in relative free cortisol levels in NCCAH patients, to better predict which patients have glucocorticoid deficiency and need glucocorticoid treatment. Until these research topics are further elucidated, clinicians should consider whether glucocorticoid treatment is beneficial for their patient based on the whole clinical picture.

## Author contributions

BA carried out the literature search, collected the included articles, carried out the initial analyses of the literature, and wrote the manuscript. MS, PS, FS, and AH reviewed and revised the manuscript. HC-G conceptualized and designed the idea of this review, contributed to the initial analyses of the literature, and reviewed and revised the manuscript. All authors contributed to the article and approved the submitted version.
